# Setting Preferences of High and Low Use River Recreationists: How Different are They?

**DOI:** 10.1007/s00267-016-0754-7

**Published:** 2016-09-06

**Authors:** Silvia Kainzinger, Arne Arnberger, Robert C. Burns

**Affiliations:** 1 Institute of Landscape Development, Recreation and Conservation Planning, Department of Spatial, Landscape and Infrastructure Sciences, University of Natural Resources and Life Sciences, Vienna, Austria; 2 Recreation, Parks and Tourism Program, Division of Forestry and Natural Resources, West Virginia University, West Virginia, USA

**Keywords:** Discrete choice experiment, Whitewater use, Boaters’ preferences, Tradeoffs, Low and high use setting

## Abstract

Whitewater boaters often choose a river based on their preferences for attributes important for their trip experience. This study explored whether preferences and tradeoffs of whitewater boaters for social, resource, and managerial attributes of riverscapes differ among a high and a low use river in the United States by employing a stated choice approach. River trip scenarios were displayed using verbal descriptions and computer-generated photographs. Results indicate that use levels were more important for boaters on the low use river, whereas river difficulty and river access fee was of higher importance for the high use river boaters, who are more involved in this whitewater activity. Preferences for waiting times and trip length did not differ between the samples. Findings suggest that whitewater boaters of high and low use rivers have a different tradeoff behavior among river setting attributes, which has implications for river recreation management.

## Introduction

Whitewater recreationists often choose a river for recreational activities based on their preferences—which are general beliefs about desirable or ideal conditions (Altman 1975)—for social, resource, and managerial characteristics of the riverscapes (Shelby and Heberlein 1986; Shelby 1980; Stewart et al. 2003; Tarrant et al. 1997). However, recreationists’ preferences are not homogenous (Ewert and Hollenhorst 1989) and may differ in recreation settings that provide different experiences. The ROS (Recreation Opportunity Spectrum) is a management framework often used to define recreation experiences (Clark and Stankey 1979). A recreation opportunity setting is defined as a combination of social, resource, and managerial conditions. Social conditions (e.g., levels and types of use) are associated with recreational use. Resource components (e.g., landscape, topography) include natural qualities, whereas managerial attributes (e.g., regulations) are provided by management (Clark and Stankey 1979). Recreationists must balance these conditions—or attributes—in their river trip choice decision making. Respondents’ choices among these recreation conditions indicate the relative importance people place on each condition and their willingness to make tradeoffs (Louviere et al. 2000). This study advances the research in tradeoff behavior by exploring whether tradeoffs and preferences for social, resource, and managerial attributes differ among whitewater boaters recreating on a low and a high use river. This is done by employing a multivariate stated choice approach which allows analyzing visitor preferences and tradeoffs among various recreation conditions.

### Preferences for Attributes Relating to River Recreation Settings

Many previous studies on whitewater recreation addressed social, resource or managerial attributes, mainly following normative and univariate approaches. However, research on preferences of whitewater recreationists have not simultaneously integrated social, resource, and managerial attributes, and has not investigated tradeoff behavior among those attributes. Past research explored preferences related to social attributes that are measures of direct impacts from too much use, such as encounters on the river, launch and rapid waiting times. Generally, boaters prefer lower use levels with few encounters (Shelby and Heberlein 1986; Shelby 1980; Stewart et al. 2003; Tarrant et al. 1997) and encounters with the same user group (Tarrant et al. 1997). Paddlers perceive encounters differently depending on the location on the river; in particular encounters at rapids are less preferred (Tarrant et al. 1997). Boaters’ enjoyment is also decreased when waiting times at rapids occur (Stewart et al. 2000). Day users are more concerned about waiting times and potentially wasting times at a boat ramp than overnight users (Whittaker and Shelby 1988).

In whitewater recreation, resource attributes such as the flow level and the number, length and difficulty of the rapids, may be a reason why boaters choose a certain river (Herrick and McDonalds 1992). Preferences for more challenging and difficult rapids are related to recreation specialization in whitewater recreation and increase with progression on the specialization spectrum (Lee et al. 2007). Further, recreation specialization is based on enduring involvement to a certain activity. Whitewater boaters with higher skill levels and a higher frequency of participation are more committed to the whitewater activity (Schuett 1993).

Managerial attributes such as the limitation and regulation of recreation use can be necessary to maintain the quality of recreation experiences (Shelby et al. 1989a). Use restrictions for whitewater recreation are a commonly used management tool in the U.S., implemented on about 110 rivers. Out of these, 15 rivers are managed with full allocation systems, restricting private and commercial use (Whittaker and Shelby 2008). An examination of hypothetical effects of river permitting procedures revealed that self-guided boaters would perceive any changes to the existing allocation system negatively, as this may lead to a reduction of trips (Siderelis and Moore 2006). Previous research has addressed preferences for social attributes, such as encounters, launch, and rapid waiting times at diverse locations at the river. However, past studies have not compared preferences for users on the river, waiting times for boat launching, and parking in one design to explore whether boaters are more affected by facility-related congestions (boat launching and parking area) or on-river congestions. It addition, past research does not provide a clear picture about whitewater boaters’ preferences for river access fees and the preferred resource length on the river for their trip.

### Different Preferences of Visitors to Low and High Use Settings

Past research identified differences in encounter preferences, acceptable use levels, and crowding perceptions between visitors recreating in low and high use density areas. The results indicate that visitors to high use areas tolerate more users than visitors to low use areas. Cole and Hall (2008), for example, found wilderness users at low use trailheads indicated preferences for lower use levels than visitors at high use trailheads. Arnberger and Eder (2009) and Arnberger et al. (2010) explored trail preferences between a low and high use forest setting in Vienna, Austria, by using the same visual trail scenarios. A comparison of these studies shows that visitors to the high use setting expressed preferences for higher use levels than visitors to the low use setting.

Previous research suggests that river users’ tolerances of recreation impacts are higher in high use density and more developed areas (Shelby 1981). Whittaker and Shelby (1988) showed that boaters in high use settings had higher acceptable levels of river encounters than boaters in low use settings. Potentially, users in higher use areas adjusted their tolerance levels for use levels more than users of low use density areas (Cole and Hall 2008).

To date, little research addressed whether preferences for social, resource, and managerial attributes differ between rivers with a similar level of difficulty, and has not compared tradeoff behavior among various river conditions among high and low use river boaters.

### Tradeoffs in Outdoor Recreation

Recent studies have addressed visitors’ tradeoffs among social, resource, and managerial attributes of recreation sites. This research is done primarily because preferred conditions might be in conflict under high level of demand, requiring visitors to make tradeoffs among these conditions (Van Riper et al. 2011). Studies addressing tradeoffs among those attributes confirmed that tradeoff behavior of recreationists in high as well as low use settings exists (Lawson and Manning 2002; Newman et al. 2005). Visitors in wilderness areas with low use levels accepted management restrictions (e.g,. regulations for camping and overnight back-country permits) over encounters with other groups (Lawson and Manning 2002; Newman et al. 2005). Similarly, recreationists on mountain summits with high use levels rated resource conditions more important and would accept management restrictions (e.g., designated trails, education signage, public access) to have less impact on the resource (Bullock and Lawson 2008; Van Riper et al. 2011). In the case of the Colorado River, whitewater boaters rated the number of encounters with other groups with higher relative importance than the time in sight of other groups during the day. The number of other groups encountered seems to be a more meaningful measure of crowding than the length of time in sight of other groups (Manning et al. 2009).

### Study Aims

Past research explored differences in preferences, norms and perceptions for visitors recreating in low and high use density settings (Absher and Lee 1981; Cole and Hall 2008; Gramann and Burdge 1984; Whittaker and Shelby 1988). However, as suggested in the ROS framework, a recreation experience is not only defined by social attributes (Clark and Stankey 1979). To date, previous research has not explicitly addressed whether tradeoffs among social (e.g., *number of people*), resource (e.g., *river difficulty*), and managerial attributes (e.g., *river access fees)* differ in river settings providing different experiences.

Stated choice modeling, including discrete choice experiments, is frequently applied to investigate tradeoffs in recreation conditions (Louviere and Timmermans 1990). In a choice experiment respondents are asked to simultaneously consider multiple alternative configurations of hypothetical, multi-attribute, goods or services (Louviere et al. 2000). Such alternatives—in our case river trip scenarios—are defined as combinations of attributes (Hensher et al. 2005). In a discrete choice experiment, two or more hypothetical alternatives are combined to choice sets and respondents choose the most, and/or least, preferred alternative from each set they are asked to evaluate. This method is rooted in the traditional micro-economic theory of consumer behavior and preference theory. Random utility theory (Louviere et al. 2000) suggests that choices can be modeled as a function of the factors of the alternatives. Selection of one alternative over the other implies that the utility of that alternative is greater than the utility of the other one. While stated choice approaches have been used for a range of recreation related issues, including whitewater recreation (Stewart et al. 2003), discrete choice experiments analyzing and comparing preferences of whitewater recreationists of a low and a high use river are very rare.

The purpose of this study was to explore whether preferences for and tradeoffs among social, resource, and managerial attributes differ between two rivers providing a different whitewater recreation experience by using a discrete choice experiment. Based on the results of previous studies we developed the following hypotheses:

H1: Boaters at a low use river setting prefer less people on the river and less waiting time for boat launching and parking than boaters at a high use river setting (social attributes).

H2: Preferences for river difficulty and trip length do not differ between two river samples because of the similar river conditions (resource attributes).

H3: Boaters recreating on a river managed with an allocation system and boaters recreating on a river without an allocation system dislike a river access fee (managerial attribute).

H4: Tradeoffs among social, resource, and managerial attributes exist for boaters in low and high use river settings (relative importance).

H5: Boaters at a low use river have lower perceived crowding ratings, report less percentage of time in sight of other groups, less acceptable percentage of time in sight of other groups, and less actual waiting times for boat launching and parking than boaters of a high use setting.

## Methodology

### Study Area

**Fig. 1:**
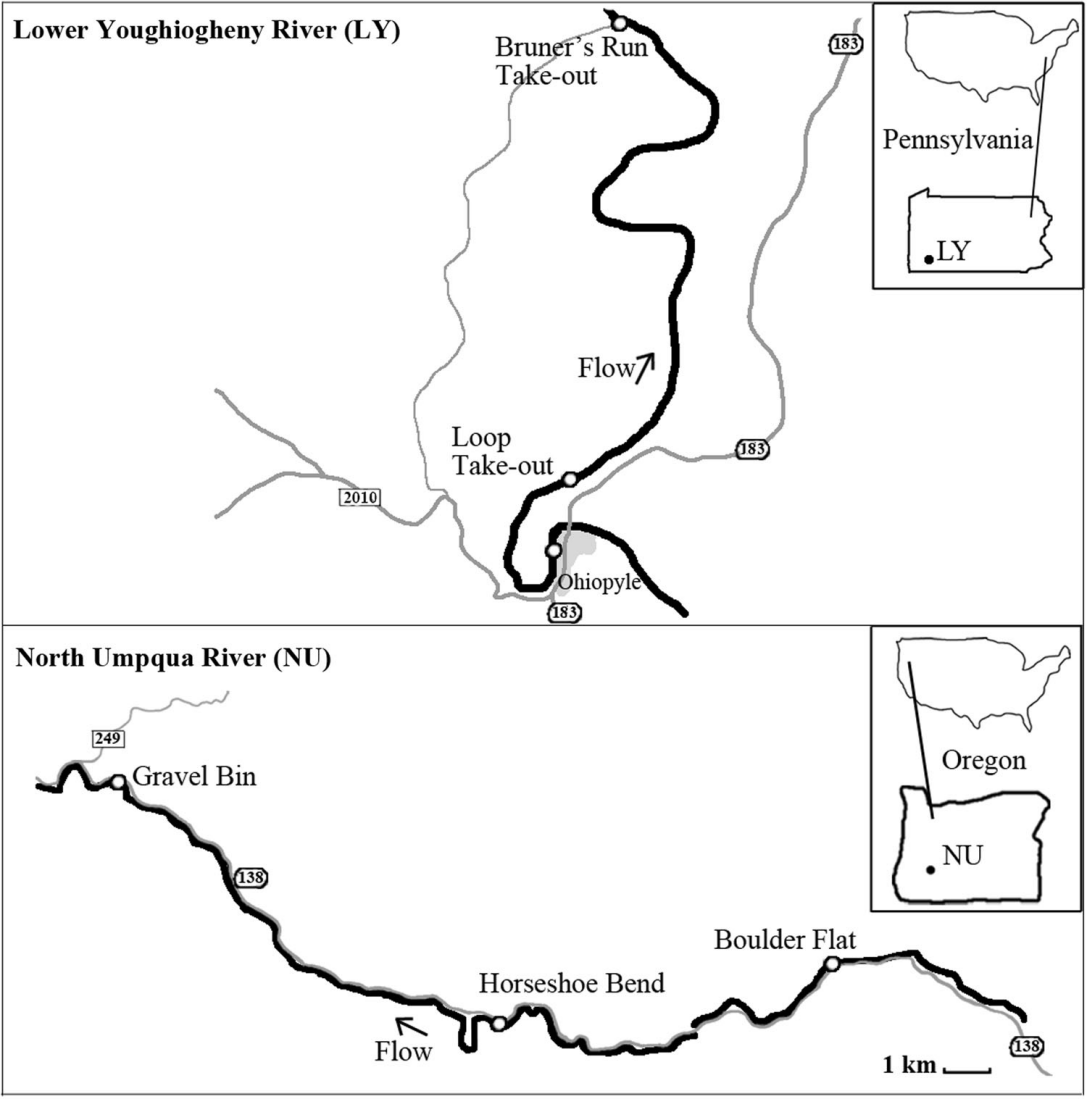
Area map of the North Umpqua River and the Lower Youghiogheny River

Data were collected on the Lower Youghiogheny ﻿River﻿, in southwestern Pennsylvania (LY) and the North Umpqua River, in southcentral Oregon (NU) (Fig. 1). These rivers were chosen as they provide a different whitewater recreation experience based on their use density and management system. Both rivers are easily accessible. The NU is in a fairly remote area with about 4.5 h driving distance from Portland, OR and about 3 h from Eugene, OR. The LY is located 70 miles southwest of Pittsburgh, PA and within about 3.5 h of several Mid-Atlantic cities, such as the Washington DC, Baltimore, MD or Cleveland, OH. The LY River access sites are located in the Ohiopyle State Park, which is very popular for various kinds of outdoor recreation such as hiking, biking, camping, and fishing. The parking lots in the Ohiopyle State Park are used by all recreation groups.

The NU is considered a low use area, as the average use per weekend during the summer was 80 private boaters (BLM and USFS 2012). The LY can be categorized as a high use area with an average of 313 boaters per weekend during the summer (Ohiopyle State Park, November 21, 2014).

The NU does not have an allocation system and a river access fee is not required. It is managed by the U.S. Forest Service and the Bureau of Land Management (BLM). The LY use is managed with a full allocation system overseen by the Pennsylvania Department of Conservation and Natural Resources (Whittaker and Shelby 2008). The number of people per day is limited to 960 commercial and 960 non-commercial passengers and the group size is limited to 25 people per group. Boaters can reserve a permit online and a waiting list system is available. This allocation system spreads out use temporally through the day via an hourly launch limit system with alternating periods of commercial and private users. Both rivers provide similar difficulty levels with at least one class IV rapid. The NU, designated as a Wild and Scenic River, offers 11 river miles and the LY 7.4 river miles for paddling. The average trip length on both rivers is four and a half hours.

### Data Sampling

The surveys were self-administered. The interviewer asked whitewater recreationists to fill out the questionnaire on-site, and took notes of group size, user types in the group and interview time. We applied the “next-to-pass-technique” (Roovers et al. 2002) and every person willing to participate in the survey was handed a questionnaire. The sample was stratified over weekdays and weekend days and time of the day. We used on-site surveys rather than mail questionnaires, presuming the data pertaining the actual experience would be most accurate when immediately recorded. The relatively low response rate can be explained by the survey location. We approached boaters at a parking lot immediately after they concluded their trip. At this time of their trip, most boaters were exhausted after hours of kayaking or rafting and were seemingly eager to depart the recreation area. A total of 601 interviews were completed with exiting users, with 203 collected at the NU and 398 at the LY River. The response rate at the NU was 43 % and at the LY 45 %. Out of the 601 interviews, 36 cases were removed because of missing values in the discrete choice experiment. Our study was limited to private boaters who were not using the service of a guide for this river trip. The small sample size and the relatively low response rate may be limitations of this study and findings therefore may not be generalized to all boater group types.

### Questionnaire on Crowding Indicators and River Preferences

The survey instrument contained questions addressing perceived crowding, percentage of time in sight of other groups, and acceptable percentage of time of seeing other groups, as well as actual waiting times for parking, put-in and take-out, socio-demographics and the rating of the personal skill level. Perceived crowding was measured using a single item, 9-point scale (Heberlein and Vaske 1977). The single-item crowding measure is easy to interpret and has been widely used in outdoor recreation research, particularly in previous studies addressing whitewater recreation. Additionally, it reduces the burden to the respondent of having to respond to multiple questions (Shelby et al. 1989b). The single-item crowding measure, however, cannot assess the perception of too few people on site and thus is not able to capture optimal conditions (Arnberger and Mann 2008).

Personal skill level was measured with two questions (Bricker and Kerstetter 2000). The interviewed rated their skill level on a 5-point scale (beginner, basic, intermediate, advanced, expert) and the difficulty of rapid class they felt comfortable to boat by themselves, ranging from class I to V. Additionally, respondents reported the total number of whitewater trips taken over the past 2 years, and the total number of whitewater trips taken on the river (Bricker and Kerstetter 2000). Enduring involvement was measured using a four dimensional approach (enjoyment, importance, self-expression, centrality) presented in eleven items on a 5-point Likert scale ranging from “strongly disagree” to “strongly agree” (Bricker and Kerstetter 2000; Schuett 1993).

**Fig. 2:**
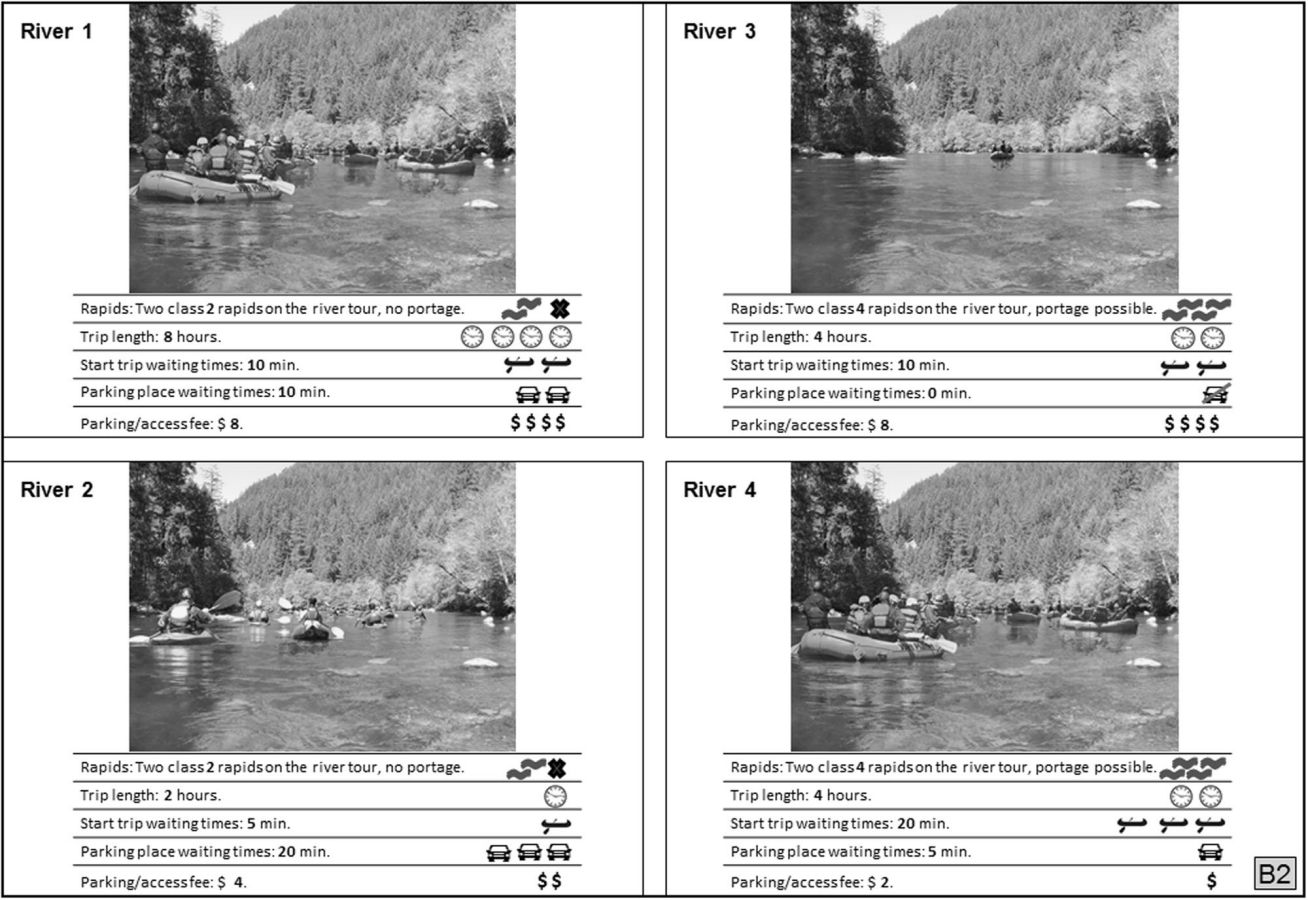
Example of a stated choice choice set. The respondents had to choose the most and least preferred choice out of four river scenarios based on the attribute levels

Each respondent evaluated a set of river trip scenarios for the discrete choice experiment (Fig. 2), with the 128 riverscape scenarios organized into 32 choice sets. Each individual evaluated four choice sets of four river scenarios, choosing the most and the least preferred scenario (Arnberger and Haider 2005). To avoid starting point bias the choice-sets were rotated systematically.

The scenarios consisted of a systematic representation of six attributes. The hypothetical river use scenarios for the discrete choice experiment were compiled by following an asymmetric orthogonal fractional factorial design plan (Addelman 1962). We selected the attributes and their levels based on existing and hypothetical conditions on the NU River. Those variables were generally applicable to whitewater recreation, important to recreationists, and representative of management concerns. The variables represent social, resource, and managerial attributes. This conceptualization is based on the ROS framework (Clark and Stankey 1979).

Three social attributes that are measures of direct impacts from too much use on the river and on land were included in the design. The social attribute *number of people on the river* presented different use levels and user types (kayakers and rafter) in eight levels. The minimum number was one kayaker and the level with the highest use level presented eight rafts with 48 people. This attribute is based on the density measure number of people at one time (PAOT) (Manning et al. 1995). Six levels of that attribute allowed a comparison of the impact of the same amount of kayaks and rafts on preferences (1, 4, or 8 kayaks vs. 1, 4, or 8 rafts). While PAOT addressed density on the river, two attributes addressed facility-related congestion; *waiting times for parking* and *boat launching*. These two 4-level attributes were defined from zero to 20 min waiting time and derived from existing conditions of the rivers under study. Since use levels on the NU River were relatively low, we did not see waiting times before rapids or for portage as major issues. However, this might be a problem at the LY River and therefore can be seen as a limitation of this study.

The resource attributes addressed the search for challenge by whitewater boaters (*river difficulty*) and their physical condition using the resource length on the river (*trip length*) as indicators. The 4-level attribute *river difficulty* ranged from two class II rapids to two class IV rapids with or without portage. *Trip length* ranged from two hours to eight hours. If the boatable section at a river is rather short (2 h), it may not be worth the effort to travel to this location. Conversely, if the section is too long (8 h) it might not be physically achievable for some boaters.

The managerial attribute is based on the fact that river use in the U.S. is often managed using allocation systems (Whittaker and Shelby 2008). The 4-level managerial variable *river access fee* was set from $2 to $8 and was calculated based on the costs of the Northwest Forest Pass. *River access fee* is also the payment vehicle in our study which estimated boaters’ willingness to pay for desired river conditions.

While this study tested a range of factors and seems to be the first to integrate social, resource, and managerial attributes in one design in the context of whitewater recreation, other variables might be of interest to test in future research. These include waiting time before rapids and portage, access time to the river resource, level of development of the setting and challenges in getting a permit.

The social attribute *number of people on the river* was displayed visually, using digitally calibrated images (Arnberger et al. 2010; Arnberger and Haider 2005; Bullock and Lawson 2008; Stewart et al. 2003; Van Riper et al. 2011), and the other five variables were presented verbally and by using pictograms. The picture used for the visual representation showed the riverscape of the NU River taken at eye level on a sunny day. The NU was used for all of the photo simulations for the following reasons. First, as a basis for comparison it was necessary to standardize scenarios for both river samples. Those scenarios focused on the recreation use variables, and the respondents were not asked to evaluate the landscape shown on the picture. Second, the picture presented a fairly neutral river landscape (even within the individual river settings, users encounter different conditions on any given day). Third, similar approaches have been successfully used in previous cross-cultural research on preferences for and acceptance of visitor use levels (Arnberger et al. 2010, Sayan et al. 2013). None of the LY respondents were irritated by the picture presented to them.

### Data Analyses

The choice model attributes were effect coded, where an *N*-categorical variable is defined by *N*-1 estimates only. Accordingly, one of the attribute levels for each attribute is the negative sum of the other level estimates. Those levels do not have standard errors, and *p*-values. Effect coding guarantees independence of all variables from the intercept, and the estimates indicate the magnitude of difference of the respective attribute level from the mean for that attribute (Hensher et al. 2005; Louviere et al. 2000). Therefore, the estimates of a multinomial logit model are all relative to each other. No base alternative or “no-choice” alternatives were presented. Therefore, no intercept exists.

To analyze the preferences of the boaters, two maximum likelihood analyses were performed with Latent GOLD Choice 4.0. This produces parameter estimates (part-worth utilities), *z*-values and standard errors for each attribute level. McFadden’s *ρ*² was used to indicate the goodness of fit of the estimated choice models, which is analogous to *R*² in ordinary regression. Values of *ρ* ^2^ between 0.2 and 0.4 are considered to be indicative of extremely good model fits (Louviere et al. 2000). Simulations by Domencich and McFadden (1975) equivalence this range to 0.7 to 0.9 for a linear function. It should not be expected that the *ρ*² values will be as high as *R*² values commonly obtained in many stated choice ordinary least square regression applications (Louviere et al. 2000). The model fit for the NU River was *ρ* ^2^ = 0.10 and for the LY River was *ρ* ^2^ = 0.09. Differences among the samples were tested using a Wald statistic test. Additionally, we calculated the relative importance of each attribute on riverscape choices per sample following the approach developed by Vermunt and Magidson (2003). Additional multivariate models were developed by adding predictors to the choice model, however none of these variables (kayaker/rafter, first time/repeat visitors, perceived crowding) were significant.

The enduring involvement items were summed to one composite variable. This index showed a Cronbach’s alpha of .933. Following the approach suggested by Shelby et al. (1989b), we split the 9-point crowding scale in two groups. One group ranged from scale points 1 to 2 indicating a positive evaluation, and the second one labeled situations where boaters reported crowding (scale points 3 through 9).

Independent and paired sampled *t*-tests and χ² tests were conducted to test for differences in socio-demographics, crowding indicators, self-reported skill level, enduring involvement and frequency of participation in the whitewater activity.

## Results

### Sample Profile

The majority of the boaters were male on both rivers. NU boaters were on average older (*M* = 42.2) than boaters on the LY (*M* = 37.6, *t* = 3.73, *p* < 0.001). Most of the boaters on both rivers indicated having at least a Bachelor’s degree. Boaters on the LY were more likely to report income higher than $99,999 (*χ* ^2^ = 13.94, *p* < 0.01). The majority of the respondents on the LY were kayaking (57.5 %), whereas more boaters on the NU were participating in rafting (53.7 %; *χ* ^2^ = 17.156, *p* < 0.001). Boaters on the LY (80.1 %) were more likely to be repeat visitors compared to the NU (67.0 %, *χ* ^2^ = 12.06, *p* < 0.001). The vast majority of the NU boaters (82.0 %) were from Oregon. Less than half of the LY boaters (41.1 %) were from Pennsylvania and the remaining from surrounding states such as Ohio (16.2 %), Maryland (9.2 %), and West Virginia (8.1 %).

### Riverscape Preferences

All six attributes predicted LY boaters’ preferences, while *waiting time for parking* and *river access fee* were not relevant for NU boaters (Table 1). The results of the samples showed some similarities. Boaters of both rivers perceived more than six people on the river negatively. Both samples disliked a *waiting time before launch* of 20 min and preferred class III and IV rapids. A four hour trip was preferred to an eight hour trip from LY and NU paddlers.

**Table 1:** Results of the choice model river sample

		North Umpqua River		Lower Youghiogheny River		
		*(n* = 203*)*		*(n* = 362*)*		
Attributes		Parameter	Std. error	Parameter	Std. error	Wald statistic
Social	*Number of people on the river*	[40.9 %]	–	[23.1 %]	–	–
	1 kayak and 1 person	0.622	–	0.386	–	–
	4 kayaks and 4 people	***0.658	0.109	***0.294	0.081	**7.194
	1 raft and 6 people	***0.735	0.107	0.145	0.077	***20.028
	8 kayaks and 8 people	−0.124	0.069	−0.023	0.052	1.385
	12 kayaks and 12 people	***−0.238	0.068	0.019	0.051	**9.122
	4 rafts and 24 people	**−0.266	0.092	−0.110	0.069	1.836
	6 kayaks, 6 rafts, 42 people	***−0.558	0.088	***−0.290	0.064	**6.058
	8 rafts and 48 people	***−0.830	0.076	***−0.421	0.055	***19.216
	*Waiting time before launch*	[12.3 %]	–	[5.9 %]	–	–
	0 min	0.202	–	0.066	–	–
	5 min	0.008	0.057	0.067	0.042	0.711
	10 min	0.061	0.057	0.006	0.041	0.598
	20 min	***−0.270	0.058	***−0.139	0.042	3.328
	*Waiting time for parking*	[3.0 %]	–	[5.3 %]	–	–
	0 min	0.022	–	0.035	–	–
	5 min	0.058	0.053	−0.021	0.039	1.427
	10 min	−0.024	0.055	*0.085	0.041	2.549
	20 min	−0.055	0.058	*−0.099	0.043	0.372
Resource	*River difficulty*	[24.7 %]	–	[36.4 %]	–	–
	2 class II rapids no portage	−0.642	–	−0.834	–	–
	2 class III rapids, no portage	**0.149	0.054	0.045	0.040	2.379
	2 class IV rapids, portage	***0.190	0.057	***0.353	0.042	*5.300
	2 class IV rapids, no portage	***0.303	0.055	***0.435	0.041	3.665
	*Trip length (time on the river)*	[14.8 %]	–	[18.1 %]	–	–
	2 h	0.003	–	0.151	–	–
	4 h	***0.247	0.058	***0.241	0.042	0.009
	6 h	0.067	0.052	−0.001	0.038	1.115
	8 h	***−0.318	0.051	***−0.391	0.039	1.302
Managerial	*River access fee*	[4.3 %]	–	[11.3 %]	–	–
	$2	0.114	–	0.198	–	–
	$4	−0.033	0.062	−0.018	0.046	0.040
	$6	−0.050	0.062	0.015	0.045	0.724
	$8	−0.031	0.056	***−0.195	0.042	*5.543

The relative importance for each attribute is displayed in parentheses

*p < 0.05, **p < 0.01, ***p < 0.001

**Fig. 3:**
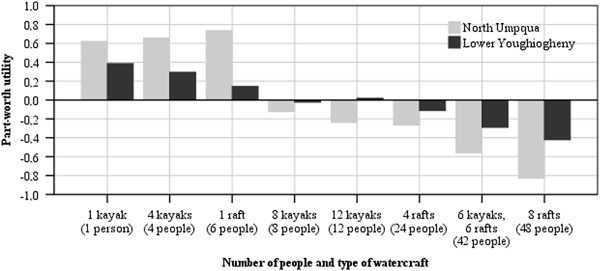
Respondents’ preferences for number of people and type of watercraft on the water

The attributes *number of peopl*e on the river, *difficulty of rapid class* and *river access fee* were significantly different between the samples. The attribute *number of people on the river* was significantly different in five levels. Paddlers of both rivers showed similar answer pattern for the *number of people on the river*, although NU boaters showed significantly higher negative part-worth utilities for very high and higher positive part-worth utilities for low *numbers of people on the river* (Fig. 3). The resource attribute *river difficulty* was significantly different in the level of two class IV rapids with portage. LY boaters showed a higher positive part-worth utility for this level than paddlers on the NU River. The managerial attribute *river access fee* was significantly different in the level with an $8 fee. LY boaters least preferred a *river access fee* of $8.

### Relative Importance of River Attributes

The attribute *number of people on the river* showed the highest importance for boaters on the NU River, whereas the *river difficulty* was the most important attribute for LY boaters (Table 1). The attribute *waiting time before launch* was more important for NU boaters, whereas the attributes *waiting time for parking*, *trip length*, and *river access fee* received slightly higher relative importance from boaters on the LY.

### Crowding Indicators and Self-reported Skill Level

All tested crowding indicators revealed differences between the river settings. Boaters on the LY indicated higher crowding (*M* = 3.72) than paddlers on the NU (*M* = 1.89). More than three-quarter of the NU boaters (77.8 %) did not report any crowding, whereas almost two thirds of the LY paddlers (61.2 %) indicated some feeling of crowding (*χ*² = 79.21, *p* < 0.001).

On average respondents on the LY reported being in sight of other groups 56.6% of the time, which is higher than the reported acceptable percentage in sight of other groups of 52.7 % (*t* = 2.42, *p* < 0.05). NU users were in sight of other groups about 15% of the time and indicated 30.6 % of the time as acceptable to see other groups (*t* = −11.43, *p* < 0.001). Boaters on the NU did not indicate waiting times for parking and for put-in and take-out, whereas paddlers on the LY reported short waiting times (Table 2).

**Table 2:** Independent sample *t*-test for crowding indicators, self-reported skill level and past experience

	North Umpqua		Lower Youghiogheny		
	*(n* = 203*)*		*(n* = 362*)*		
	Mean	Std. dev.	Mean	Std. dev.	*t*-value
*Crowding indicators*					
Perceived crowding^a^	1.89	1.32	3.72	2.17	−12.45***
Percent of time in sight of other groups	15.02	16.48	56.29	30.00	−20.99***
Acceptable percent of time seeing other groups	30.64	18.48	52.42	24.62	−11.83***
Waiting time for parking (min)	0.44	1.90	3.60	15.34	−3.87***
Waiting time for boat launching (min)	0.84	3.37	3.62	9.77	−4.89***
Waiting time at take-out (min)	0.51	2.66	2.76	6.38	−5.83***
Self-reported skill level^b^	3.23	1.12	3.27	1.12	−0.40
Class of difficulty without the service of a guide^c^	3.93	0.92	3.87	0.93	0.70
Number of total whitewater trips taken in the past 2 years	21.23	38.52	43.57	74.35	−4.68***
Number of whitewater trips taken to this river in the past 2 years	4.30	10.72	13.80	27.72	−5.76***
Enduring involvement^d^	3.82	0.92	4.05	0.73	−3.10**

***p < 0.001, **p < 0.01

^a^ Scale: 1, 2 = not at all crowded; 3, 4 = slightly crowded; 5, 6, 7 = moderately crowded; 8, 9 = extremely crowded

^b^ Scale: 1 = inexperienced/beginner/novice; 2 = some experience/basic; 3 = intermediate; 4 = advanced; 5 = expert

^c^ Scale: 1 = class I; 2 = class II; 3 = class III; 4 = class IV; 5 = class V

^d^ Scale: 1 = strongly disagree, 5 = strongly agree

The self-reported skill level was not significantly different between the two samples and boaters rated themselves as intermediate and able to run at least a class IV rapid. Boaters on the LY reported a higher number of total whitewater trips taken in the past 2 years (*M* = 43.57) than boaters on the NU (*M* = 21.23). LY boaters took more trips on this river (*M* = 13.80) than NU boaters (*M* = 4.30). Further, LY paddler were more committed to the whitewater activity (*M* = 4.05) than NU-boaters (*M* = 3.82).

## Discussion

This study found that social, resource, and management-related attributes influenced boaters’ preferences and that differences between the samples for several of these attributes exist. Results indicated that *the number of people on the river* were more important for boaters on the low use river, whereas *river difficulty* and *river access fee* were of higher importance for boaters recreating in a high use river setting. Preferences for *waiting time before launch *and *waiting time for parking* and *trip length* did not differ between the samples. Findings suggest that whitewater boaters of high and low use rivers have a different tradeoff behavior among river setting attributes.

### Different Preferences for River Attributes

The differences between the samples originated in the lower or higher part-worth utility values of one social attribute (*number of peopl*e), one resource attribute (*river difficulty*), and the managerial attribute (*river access fee*). Respondents in both of the samples showed similar patterns for support or dislike, however certain attributes and attribute levels elicited a stronger response from the boaters of different river settings and therefore were relatively more important. This information is useful to evaluate the importance visitors place on particular aspects of outdoor recreation experience, which can help to understand trip choice decision making (Louviere and Timmermans 1990). The results of the attribute *number of peopl*e confirm that boaters choose a specific site based on their preferences for use levels (Shelby et al. 1983). Therefore, hypothesis H1 can be confirmed in the context of that attribute. Our data supported previous findings that indicated that boaters have preferences for lower use levels (Shelby and Heberlein 1986; Shelby 1980; Stewart et al. 2003; Tarrant et al. 1997), that rafts were less preferred than the same amount of kayaks (Tarrant et al. 1997) and that boaters recreating in a low use density setting preferred a lower *number of people on the river* than paddlers in a high use density area (Cole and Hall 2008). However, our results also revealed differences in the preferred *number of peopl*e *on the river* between the two settings. While LY users’ preferences linearly decreased with increasing river user numbers, the NU boaters’ parameter values sharply dropped when more than one raft or 4 kayaks were visible (Fig. 3). NU paddlers desired social condition includes the company of a few small other groups, but they strongly disliked medium and high use levels, while LY boaters appear to accept the presence of others to a greater degree.

Boaters of both rivers showed similar preference patterns for the resource attribute *river difficulty,* confirming hypothesis H2 in the context of that attribute. Both rivers provide a similar difficulty level and therefore attract boaters with similar skills, since no difference in reported skill levels was found. However, the attribute *river difficulty* was most important for LY boaters, while the *number of people on the river* was the most important attribute for NU boaters. Thus tradeoffs exist among the attributes confirming the hypothesis H4 and differ between low and high use rivers. Those results indicate that boaters recreating in a high use setting are more willing to tolerate higher numbers of users to achieve the desired resource condition (*river difficulty*) than paddlers in a low use setting. Setting attributes play an important role in whitewater boaters’ trip evaluation (Herrick and McDonald 1992), and many boaters are motivated by challenge (Galloway 2012). LY paddlers were younger, mainly kayakers, who participated more frequently in and are more committed to the activity (Schuett 1993). We suggest that those paddlers focus more on the activity itself and are less concerned about crowded situations. In addition, LY boaters have less substitute rivers available in close proximity than NU boaters have in Oregon, and must accept the existing conditions. River use regulations are used commonly on rivers in the U.S. (Whittaker and Shelby 2008) and it is therefore in the interest of managers to better understand whitewater boaters’ preferences for management actions. A fundamental question managers of whitewater recreation resources often face is between providing access to the river and management restrictions to protect specific recreation opportunities. A frequently used management approach is a *river access fee*. This study indicates that LY boaters (using a river with a full allocation system) place a higher importance on *river access fees* than NU boaters, who were recreating on a river without restrictions. LY boaters preferred a *river access fee* of $2 and a higher fee was negatively perceived. The fact that the attribute *river access fee* was only significant for LY boaters only partially confirms the hypothesis H3. The frequency of participation may explain the different results for the attribute *river access fee*. LY boaters participate more frequently in whitewater activity, and are more involved in whitewater recreation than boaters of the NU. A change of the existing allocation system (Siderelis and Moore 2006) or an increase in *river access fee* could potentially mean a decrease in participation in that activity for them. Our findings suggest that boaters recreating on the NU River are willing to accept management restrictions (such as a *river access fee* up to eight dollars) to achieve the desired social (*number of people on the river*) and resource (*river difficulty*) conditions. However, a river access fee higher than eight dollars might have an impact for NU boaters as well.

### Similar Preferences for River Attributes

The samples had similarities regarding their preferences for two social and one resource attribute. Therefore we could only partially accept the hypotheses H1 and H2. Preferences for *waiting time at the boat launch* were significant but did not differ between the samples. This attribute is associated with density at the access area of the river and density at access areas, and is of particular managerial interest (Gramann and Burdge 1984). Paddlers in our study were typically day users, and therefore might be concerned about wasting time at the boat ramp (Whittaker and Shelby 1988). Boaters may also fear that another group blocking the boat ramp might lead to crowding-related congestions on the water. However, the direct contact and queuing at a boat ramp with potentially limited space available do not seem to be very important concerns for boaters of either river, in particular for LY users. The actual reported numbers of waiting times at the boat ramp in our samples were very low and confirm that waiting times are not a very relevant issue in both cases.

The attribute *waiting time for parking* had small effects on boaters’ preferences, and only long waiting times (more than 10 min) were significant for LY boaters. There are other factors which were more relevant to paddlers, such as crowding indicators on the water and resource related factors (Herrick and McDonald 1992). Regardless of the use density on the water, it seems like boaters are less concerned about waiting times at the parking lot or boat ramp. This finding shifts the emphasis from facility-related congestions to on-river social issues. Boaters seem to be more affected by crowding indicators that are directly associated with the leisure activity such as *number of people* on the water than *waiting time at the boat launch*. Future research may examine whether waiting times at rapids is of higher importance than waiting times at boat ramps and parking lots.

Preferences for the resource attribute *trip length* were similar across the samples. A *trip length* of four hours was most preferred, and eight hours were disliked. Both rivers provided a river experience with similar trip length and did not offer multiday trips with camping opportunities on the river. The majority of the NU boaters were from Oregon, whereas the LY boaters came mainly from Pennsylvania or the bordering states of Ohio and West Virginia. Accordingly, they did not travel a great distance to access the river. It appears that a four hour trip is a reasonable timeframe for boaters taking day trips to an intermediate whitewater river setting.

### Crowding Indicators

All crowding indicators differed between the low and the high use river setting, confirming hypothesis H5. NU boaters have lower thresholds and are less tolerant of other visitors on the river than LY paddlers. Crowding seems not to be at a level of concern for management of the NU River, since more than two thirds of the boaters did not perceive any crowding (Shelby et al. 1989b). Tolerances for NU boaters’ use level preferences were not exceeded; which was also reflected in their low crowding ratings. Although LY paddlers are used to higher densities, our findings show that about more than half indicated some level of crowding. They did not encounter their preferred social conditions and therefore, their tolerances have been exceeded (Shelby et al. 1983). Crowding levels in this range indicate that social carrying capacity limits are almost reached (Shelby et al. 1989b).

## Management Implications and Conclusions

This study advances the research in non-motorized whitewater boaters’ preferences for social, resource and managerial attributes by integrating all in one design and comparing preferences between two settings. The two river settings provided a different whitewater experience because of the different use densities and management approaches. We found that whitewater boaters’ preferences and tradeoff behavior for *number of people on the river*, *river difficulty* and *river access fee* differed between the rivers. Even though the response patterns of the preferred levels were similar for the samples, results differed in the part-worth utilities and therefore relative importance placed on attribute levels. Boaters at the low use river placed more importance on the social attributes of river use, while the resource attribute *river difficulty* and the managerial attribute *river access fee* were more important for high use river users, who are more committed to that whitewater activity.

The results of the discrete choice experiment have implications for the ROS framework by showing which of the three realms (social, resource, or managerial attributes) plays a larger role in attracting potential use. This knowledge can help resource managers to better target a specific group of whitewater boaters based on resource availabilities. It seems to be useful that managers identify the recreational role of their resource in a specific area, instead of planning and managing for all possible recreation opportunities (Aukerman and Haas 2004). However, due to the small sample size and the sampling method, our results show only tendencies for low and high use river settings in the U.S., and further research is necessary for confirmation.

One management implication of our findings is that boaters of the NU would tolerate management restrictions. NU River management can ensure low use levels in the future by using direct management systems. These may include user fees up to eight dollars, or may consider the establishment of partial allocation systems, negotiated calendars for launches or restricting the number of launches per day. Boaters of a river setting which provides a unique experience based on its difficulty or location might desire to paddle on this river regardless of the amount of people. High river access fees, however, could lead to reduced visitation, as the significant influence of the *river access fee* attribute for LY users has shown.

Overall the management approach on the NU River seems to be working well, and if use numbers remain low there is no need to change the current system. The management approach on the LY might already be at its capacity limits, as our results indicated that many LY paddlers reported crowding. The allocation system at the LY is designed to spread out use temporally through the day via an hourly launch limit system with alternating periods for commercial and private users. However, if groups decided to take breaks during their trip there is potential that they might encounter the group that was launching, evoking crowding perceptions. Extending the time slots between the groups might provide a better experience for whitewater boaters on the LY.

This study concentrated on whitewater boaters’ preferences for social, resource, and managerial attributes in two different rivers in the United States. To gain a better understanding of the heterogeneity of preferences for these attributes, further research in other outdoor recreation activities is needed. Additionally, to further enhance the research in tradeoff behavior of recreationists it is necessary to explore recreation groups other than whitewater boaters, such as wilderness users, and mountain summit users in high and low use settings.
